# Fluorescence Imaging of Actin Turnover Parses Early Stem Cell Lineage Divergence and Senescence

**DOI:** 10.1038/s41598-019-46682-y

**Published:** 2019-07-17

**Authors:** Prakhar Mishra, Daniel C. Martin, Ioannis P. Androulakis, Prabhas V. Moghe

**Affiliations:** 1 Cell and Developmental Biology graduate program in Molecular Biosciences, Rutgers University, Piscataway, NJ 08854 USA; 2 Department of Biomedical Engineering, Rutgers University, Piscataway, NJ 08854 USA; 3 Department of Chemical and Biochemical Engineering, Rutgers University, Piscataway, NJ 08854 USA

**Keywords:** Stem-cell biotechnology, Lineage tracking

## Abstract

This study describes a new approach to discern early divergence in stem cell lineage progression via temporal dynamics of the cytoskeletal protein, F-actin. The approach involves real-time labeling of human mesenchymal stem cells (MSCs) and longitudinal tracking of the turnover dynamics of a fluorogenic F-actin specific probe, SiR-actin (SA). Cells cultured in media with distinct lineage factors and labeled with SA showed lineage specific reduction in the actin turnover shortly after adipogenic (few minutes) and chondrogenic (3–4 hours) commitment in contrast to osteogenic and basal cultured conditions. Next, composite staining of SA along with the competing F-actin specific fluorescent conjugate, phalloidin, and high-content image analysis of the complementary labels showed clear phenotypic parsing of the sub-populations as early as 1-hour post-induction across all three lineages. Lastly, the potential of SA-based actin turnover analysis to distinguish cellular aging was explored. *In-vitro* aged cells were found to have reduced actin turnover within 1-hour of simultaneous analysis in comparison to cells of earlier passage. In summary, SiR-actin fluorescent reporter imaging offers a new platform to sensitively monitor emergent lineage phenotypes during differentiation and aging and resolve some of the earliest evident differences in actin turnover dynamics.

## Introduction

Mesenchymal stem cells (MSCs) have been widely used for their ability to self-renew and differentiate into cells of distinct lineages including adipocytes, osteoblasts, and chondrocytes^1^. As a cell type that can be harvested from multiple sources and are easily expanded *in-vitro*, MSCs have become a valuable tool for experimental and clinical cell based regenerative applications^2^. One of the challenges for using cells with multi-lineage potential is the ability to track and control their lineage-specific differentiation. Given the possible heterogeneity that a population of MSCs adopts, methods to distinguish single cell lineage staging within a population would be extremely advantageous for selecting cells that have potential utility in regenerative medicine.

MSC differentiation is accompanied by a significant degree of reorganization of the intracellular cytoskeleton^3–6^. Both the dynamics and architecture of the actin network play a vital role in differentiation^6^. Disrupting actin cytoskeleton by cytochalasin D (actin polymerization inhibitor) results in reduced osteogenesis but increased adipogenesis and chondrogenesis^5,7^. Sliogeryte *et al*. reported that MSC differentiation led to overall increase in actin organization and slower turnover after 7 days of induction^8^. Actin cytoskeletal organization and dynamics play a determining role in regulating MSC differentiation and could be used as a marker for assessment of stem cell behavior. Treiser *et al*. used organizational features of F-actin to forecast emergent MSC lineages based on high-content image analysis^9^. Despite the new insights these reports reveal about the heterogeneity of the cytoskeleton in lineage diverging culture conditions, these studies were based on phalloidin staining of fixed cells, and thus were largely restricted to static comparisons of cell phenotypes and long-range shifts in these phenotypes (over days). Some of the earliest known changes in the cytoskeletal morphology during MSC differentiation to date have been described as early as 24 hours post-induction even with flow cytometric analysis^9,10^. Little is known about the dynamic changes in the actin cytoskeleton during the initial, emergent stages of lineage divergence. We propose that early reorganization of actin precede the lineage specific morphological changes. Therefore, the dynamics of the actin network could act as a sensitive marker of lineage divergence.

MSCs require *in-vitro* expansion to generate adequate cell numbers for most clinical or research-based applications. Unfortunately, MSCs are susceptible to senescence during prolonged cell culture leading to reduced proliferation, and differentiation potential^11^. MSC aging also results in altered expression of actin-associated proteins and decreased actin turnover^12^. Similar to MSC differentiation, actin reorganization plays a role in aging as well, therefore it is important to elucidate the kinetics of actin turnover in both differentiation and senescence phenomena.

**Figure 1:**
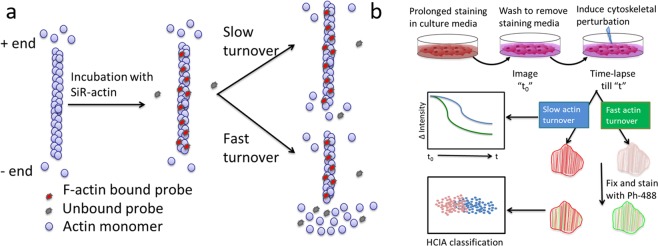
Probing altered actin dynamics in live-cells based on an F-actin specific fluorogenic probe. (**a**) Schematic showing F-actin interaction with SiR-actin (SA), a cell permeable probe that fluoresces when bound to F-actin. Actin monomers are assembled/disassembled at either ends of the filament with different rates creating the fast growing “+” end and the slow growing “−” end. SA binds F-actin with high specificity, but upon removal from the filament due to actin turnover, the probe loses >100 fold in fluorescence. Probe binding is inversely correlated with actin turnover^14^. (**b**) SiR-Actin based Measurement of Actin Turnover (SMAT) involves prolonged live cell staining in the growth media with SA, removal of staining media, addition of cytoskeleton influencing cues and simultaneous time-lapse imaging. The loss of SA due to altered actin dynamics is quantified via the ratiometric decay of intensity in the image series captured during time “t” and normalized to the first timepoint (t_0_). Further, subtle phenotypic variations in the actin cytoskeleton are parsed via high content image analysis (HCIA), based on dual imaging of SA and F-actin specific fluorescent probe, phalloidin (ph-488).

In this study, we employed an F-actin specific cell permeable probe, SiR-actin (SA), for real-time assessment of the kinetics of actin turnover during early stages of differentiation and cellular aging. The highly dynamic actin filaments undergo the addition or removal of the monomers (G-actin) at unequal rates on either end (Fig. 1a). The addition of G-actin is favored at the more dynamic plus end, while the monomers are turned over at the minus end^13^. Milroy *et al*. proposed that SA binds F-actin only at the sites with three contiguous G-actin monomers, and the rate of actin turnover is faster than the rate of SA binding. Therefore SA incorporation is favored at the F-actin sites with slow dynamics (Fig. 1a)^14^. Detachment of SA from its binding site due to filament turnover results in 100-fold decline in the fluorescence^15^. Because of this property, the highly dynamic actin structures (such as lamellipodia or filopodia) are weakly stained by SA while the more complex and long lived structures (such as ventral stress fibers and transversal arcs) are strongly labeled with SA (Fig. 1a)^14^.

The key premise for this paper is that distinct actin turnover rates can be resolved and quantified through imaging of SiR-actin probe binding behaviors, a method we call SiR-actin based measurement of actin turnover (SMAT). The SMAT workflow (Fig. 1b) involves prolonged incubation of cells in SA supplemented growth media, wash/removal of the staining media, introduction of the cytoskeleton-perturbing cue, time-lapse epifluorescence/confocal fluorescence imaging and image processing and data analysis and modeling. Change in actin turnover exhibits altered SA staining, which can be measured as a function of time by ratiometric intensity plot of the image series. Morphological reorganization of the cytoskeleton can be further benchmarked against F-actin specific probes, such as Phalloidin (P). SA stained cells (red) with more dynamic F-actin regions offer more binding sites to enable P binding (green) and show increased green color intensity compared to the less dynamic regions. This dual SiR-actin-Phalloidin (S-P) imaging protocol was used in conjunction with high-content image analysis to elucidate temporal lineage specific alterations in actin cytoskeleton during lineage progression (Fig. 1b).

The focus of this paper was to first calibrate SMAT with cytoskeletal drugs to validate actin turnover dependent loss of SA staining. Next, we characterized and quantitatively modeled the temporal profiling of MSC lineage divergence to identify actin turnover with lineage specific kinetics. We also developed a composite staining framework with competing F-actin probes to further profile, via high content imaging, minute phenotypic changes in the actin cytoskeleton underlying the lineage specific changes. Lastly, as a potential application to regenerative medicine, SMAT was deployed to discern between later and earlier passaged cells.

## Results

### Validation of SiR-Actin Labeling for quantification of actin turnover with cytoskeleton-perturbing drugs

**Figure 2:**
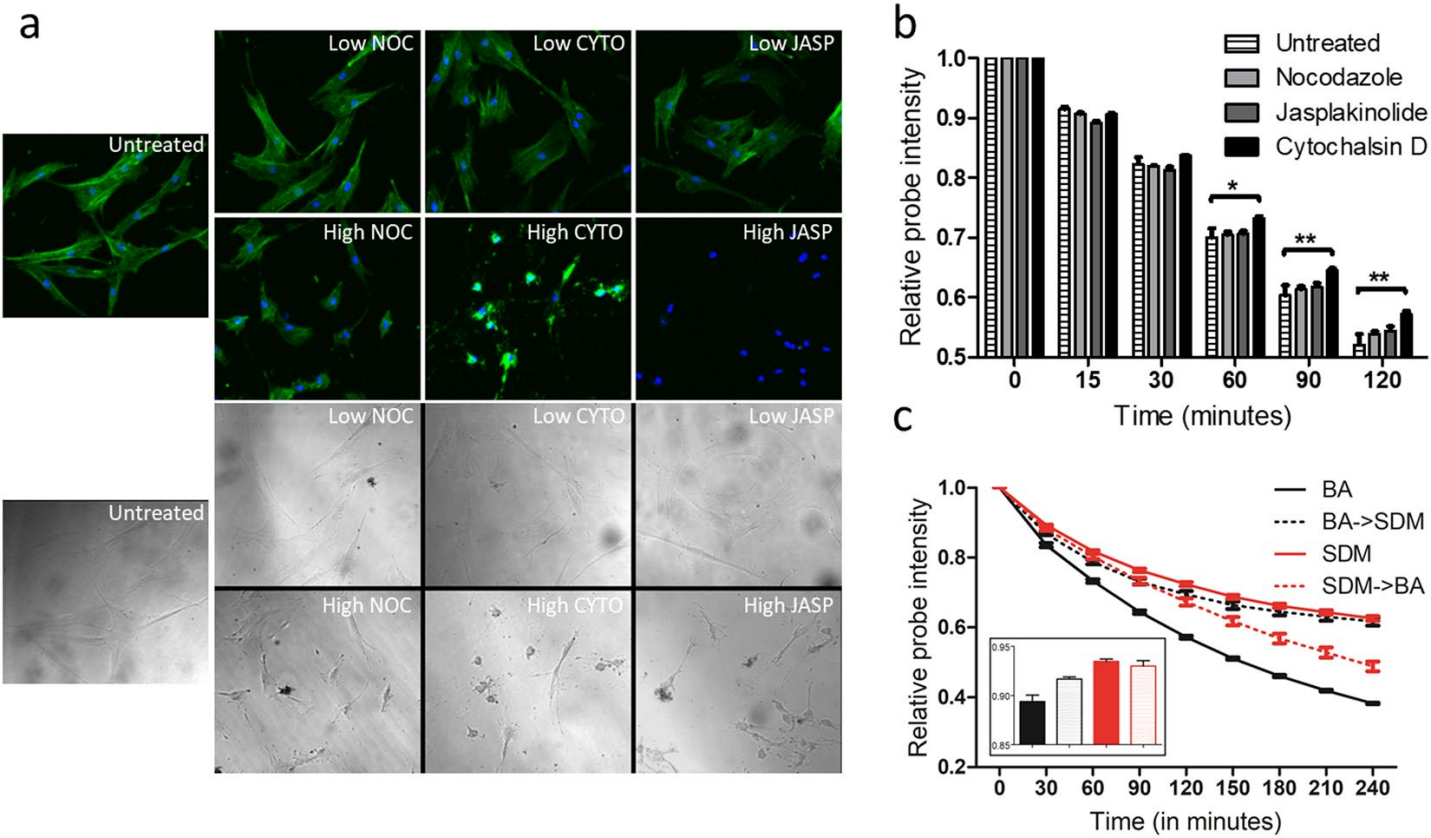
Characterizing the influence of cytoskeletal perturbations on SiR-actin staining. (**a**) Change in actin morphology 2 hrs of treatment with low or high dose of drugs. Conditions include: untreated, nocodazole- (8 nM, 8uM; NOC), cytochalasin D (1 nM, 1 uM; CYTO) and jasplakinolide (1 nM, 1 uM; JASP). (**b**) Change in SA staining due to altered actin turnover by low dose of cytoskeletal drugs. For each group, values are mean + standard error (n = 3). *p < 0.05, **p < 0.01, ***p < 0.001 vs the untreated group. (**c**) Change in SA staining due to serum deprivation. BA: basal media, SDM: serum deprived media, BA- > SDM: SA staining performed in BA, then switched to SDM, SDM- > BA: SA staining performed in SDM, then switched to BA. Inset: SA intensity at 15 minutes. For each group, values are mean ± standard error (n = 3). ***p < 0.001 for BA vs SDM, ***p < 0.001 for SDM- > BA vs SDM, *p < 0.05 for BA vs BA- > SDM, n.s. for BA- > SDM vs SDM after 120 min.

The cytoskeletal drugs with known effects on actin dynamics were used to validate SiR-Actin labeling. Initially, the effect of cytoskeletal drugs on actin morphology was investigated. The three drugs were cytochalasin D (CYTO, actin polymerization inhibitor), nocodazole (NOC, microtubule polymerization inhibitor) and jasplakinolide (JASP, actin polymerization promoter). Typically, MSCs are elongated spindle-shaped with defined stress fibers. SA stained cells treated with the high dose of cytoskeletal drugs demonstrated distorted actin cytoskeleton after two hours (Fig. 2a). Treatment with 8 uM NOC diminished the cell size, but the SA staining was intact and appeared similar to untreated cells. Both 1 uM CYTO and 1 uM JASP treatments disrupted the actin cytoskeleton to an amorphous mass with no stress fibers. However, CYTO treated cells preserved the SA stain in the collapsed cytoskeleton, while JASP treated cells lost the SA staining entirely (Fig. 2a). This pronounced effect on SA staining stems from the differences in the mechanisms of action of the two drugs. CYTO induced actin cytoskeleton collapse preserved the SA bound on actin filaments, whereas JASP, owing to its structural similarities to SA, liberated the probe during cytoskeletal collapse. These observations provided the evidence for actin dynamics-dependent SA staining.

In order to test the sensitivity of the SMAT pipeline, very small doses of cytoskeletal drugs were identified to alter actin turnover without any apparent changes in the cell shape. For SMAT analysis, the lowest drug concentrations that led to no gross changes in actin morphology were 1:1000 dilution of the drugs (1 nM CYTO, 1 nM JASP, 8 nM NOC) (Fig. 2a, Supplementary Fig. 1.). 1 nM CYTO showed slower decay of SA intensity which emerged after 30 minutes of treatment, then reached significance after 1 hour (p < 0.05), while JASP and NOC showed no statistical significance compared to untreated cells (Fig. 2b). These results are in agreement with the observations in Fig. 2a with 1 uM CYTO treatment, because 1 nM CYTO interfered with actin turnover resulting in prolonged SA staining. Interestingly, SMAT analysis discerned changes in the actin turnover caused by low dose of cytoskeletal drugs even with no apparent changes in actin morphology (Supplementary Fig. 1).

As another approach to explore our workflow for dynamic assessment of actin turnover, SMAT was performed with cells cultured in serum deprived media (SDM) (Fig. 2c). Serum depletion during cell culture is known to induce cell growth arrest along with actin depolymerization^16^. Indeed, SMAT analysis showed that cells in SDM showed significantly slower SA decay compared to those cultured in complete basal (BA) media (SDM vs BA: p < 0.001 at 30 min). The serum induced growth arrest can be reversed by reintroduction of serum in the culture media, causing increased actin polymerization^16^. Reintroducing serum containing BA in cells (SDM- > BA), resulted in rapid decline in SA intensity due to accelerated actin turnover (SDM vs SDM- > BA, p < 0.001 at 120 min). Similarly, initiation of serum deprivation showed concurrent reduction in SA decay rate (BA- > SDM vs BA, p < 0.05 at 30 min). High sensitivity of SMAT to the changing actin turnover was demonstrated by early emergence of differences in SA intensity among the groups as early as 15 minutes (Fig. 2c, inset). In summary, SMAT analysis demonstrated the ability to monitor dynamics of actin turnover.

### Live-profiling of differentiation induced cytoskeletal reorganization

**Figure 3:**
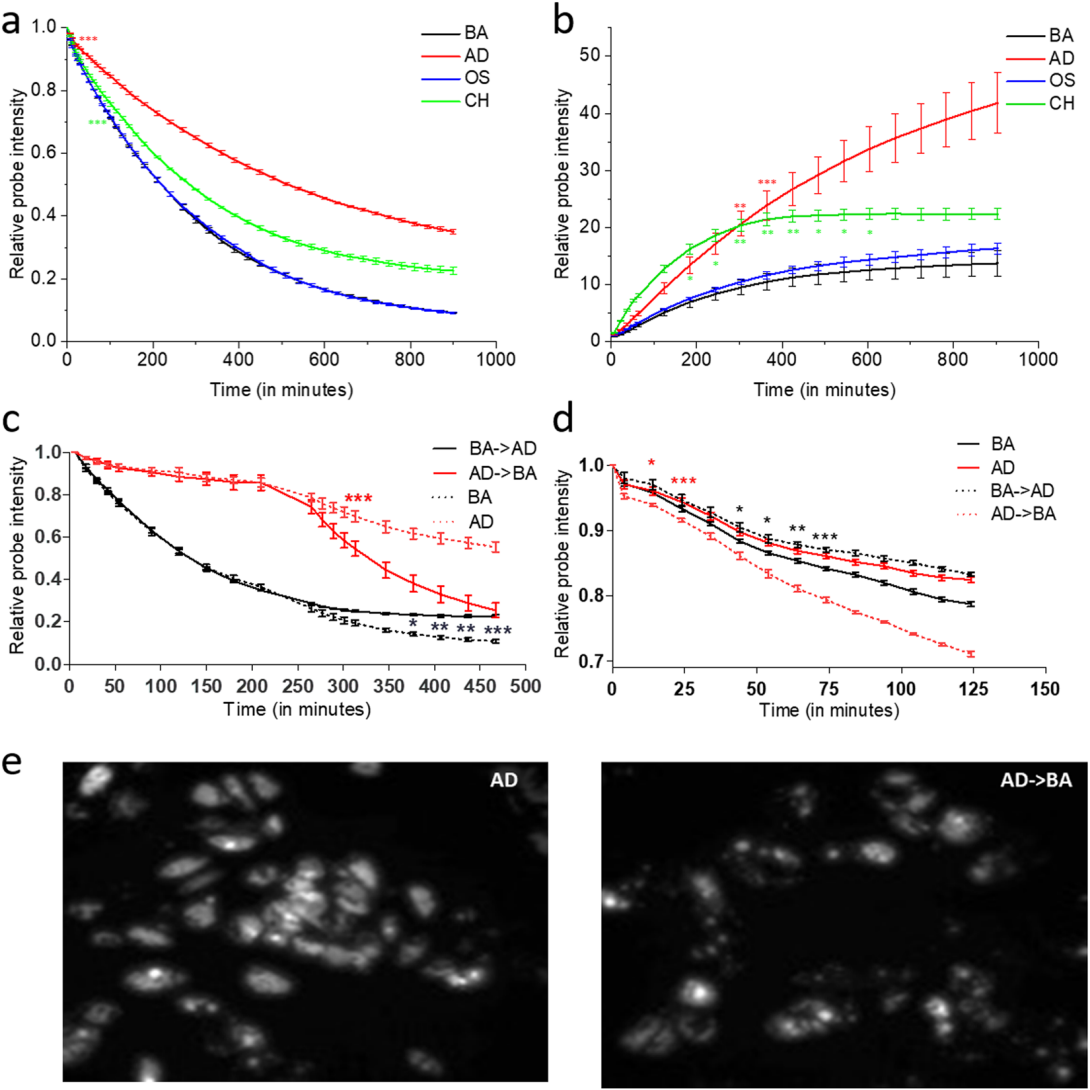
Temporal profiling of actin turnover in MSCs in response to differentiation induction. (**a**) SMAT analysis enabled early parsing of MSCs exposed to adipogenic (AD), chondrogenic (CH), and osteogenic (OS) from basal (BA) growth media. For each group, values are ratiometric mean ± standard error of SA intensity normalized to the first timepoint within the respective group. *p < 0.05, **p < 0.01, ***p < 0.001 vs the BA group (n = 3). (**b**) Alternative approach to SMAT with unstained MSCs showed distinguished rates of increase in SA intensity when introduced with BA, AD, OS and CH media. The statistical analysis was done similar to A. (**c**) SMAT analysis showed temporal sensitivity of cytoskeleton during media switch. After 4 hours of initial induction with AD, media swap resulted in switched probe decay profiles within minutes. For each group, values are ratiometric mean ± standard error of SA intensity normalized to the first timepoint within the respective group. P values were calculated after the media switch. *p < 0.05, ***p < 0.001 BA vs BA- > AD and **p < 0.01, ***p < 0.001 BA vs BA- > AD group (n = 3). (**d**) SMAT analysis demonstrates cytoskeletal plasticity after lineage commitment: after 15 days of adipogenic induction switching to BA resulted in faster SA decay. P values were calculated similar to C. (**e**) Adipored stained cells showed a reduction in the number of adipocytes after 3 days of media swapping from AD to BA (15 + 3 days).

MSC differentiation invokes extensive cytoskeletal reorganization in response to the soluble cues^6^. Previously, our lab demonstrated that higher order organizational features of actin cytoskeleton could be used to distinguish cells in adipogenic or osteogenic media after 72 hours of initial exposure^9^. We hypothesized that evaluation of kinetics of actin turnover via SMAT would enable faster temporal resolution of distinct cell fates. SA-stained cells in basal growth media (BA) were stimulated with adipogenic (AD), osteogenic (OS) or chondrogenic (CH) media and subsequently imaged for ~15 hrs. All soluble cues demonstrated a unique SA decay profile due to slow down of actin turnover albeit at different times (Fig. 3a). AD demonstrated slowest probe decay compared to all other conditions and reached significance within minutes, followed by CH and OS (Fig. 3a, Supplementary Video 1). Therefore, SMAT enabled parsing the live MSCs undergoing differentiation within minutes to few hours.

The SMAT approach entails SA based dissociation (off-rate) and imaging-based profiling. We also undertook a complementary SA association kinetics approach by initiating the live-imaging with unstained cells and subsequent addition of BA, AD, OS and CH supplemented with SA. Here, the increase in SA staining indicates slower actin turnover while the dynamic actin sites are poorly stained as mentioned earlier. Similar to SMAT analysis, the SA kinetics followed an exponential behavior. Again, AD showed the slowest actin turnover, with more rapid kinetics in CH and OS conditions (Fig. 3b, Supplementary Video 2). The SA kinetics showed variations and did not resolve the trendlines for OS and CH from BA. Therefore, the dissociation kinetics approach in SMAT was found to be more sensitive for resolving changes in actin turnover.

#### Determining temporal responsiveness of actin cytoskeleton by switching soluble cues

Initiation of differentiation leads to extensive changes in the cytoskeletal organization. But the cytoskeletal plasticity during the course of MSC differentiation is not well understood. Next, we explored the temporal responsiveness of the cytoskeleton to the lineage specific cues by swapping the medias with concurrent SMAT analysis. SA stained cells were induced with AD for 3.5 h, which showed similar trends as seen previously (Fig. 3a). The media was swapped at 4 h timepoint and imaging was done from 4 to 8 h. The switch from AD- > BA showed significantly faster SA decay compared to the AD group (p < 0.01 after 32 minutes of media switch), while BA- > AD switch showed slower actin turnover vs BA (p < 0.05 after 120 minutes of media switch) (Fig. 3c). Also, SMAT analysis remained sensitive to the change in actin turnover over the course of this study, particularly for BA vs BA- > AD group, which had already lost most of the SA at the media switch.

A similar longer-term media swap experiment and SMAT analysis was done after 14 days of adipogenic induction. Several cells with intracellular lipid droplets were seen under the bright field microscope in AD (data not shown), while cells in BA were mostly elongated and spindle shaped. At Day 14, cells were stained with SA and the media switch was performed on the following day. BA- > AD showed significantly slower SA decay compared to the BA group (BA vs BA- > AD p < 0.05 at 44 min), while AD- > BA showed faster SA decay compared to the AD group (p < 0.05 at 14 min) (Fig. 3d). The rapid response from BA- > AD and AD- > BA groups shows that the cytoskeleton remains plastic and responds instantly to the soluble cues even in lineage committed cells. After the media switch, cells were cultured for 3 more days. On 18^th^ day, the cells were fixed and stained with adipored. BA- > AD group showed emergence of lipid droplets and AD- > BA group showed reduction in the number of adipocytes compared to the AD group (Fig. 3e). While other groups have described dedifferentiation for MSCs^17^, here we have demonstrated that the actin cytoskeleton remains plastic and responds rapidly to the lineage specific soluble cues, even with lineage committed MSCs.

### F-actin composite staining based high content image analysis for assessment of morphological changes during lineage progression

**Figure 4:**
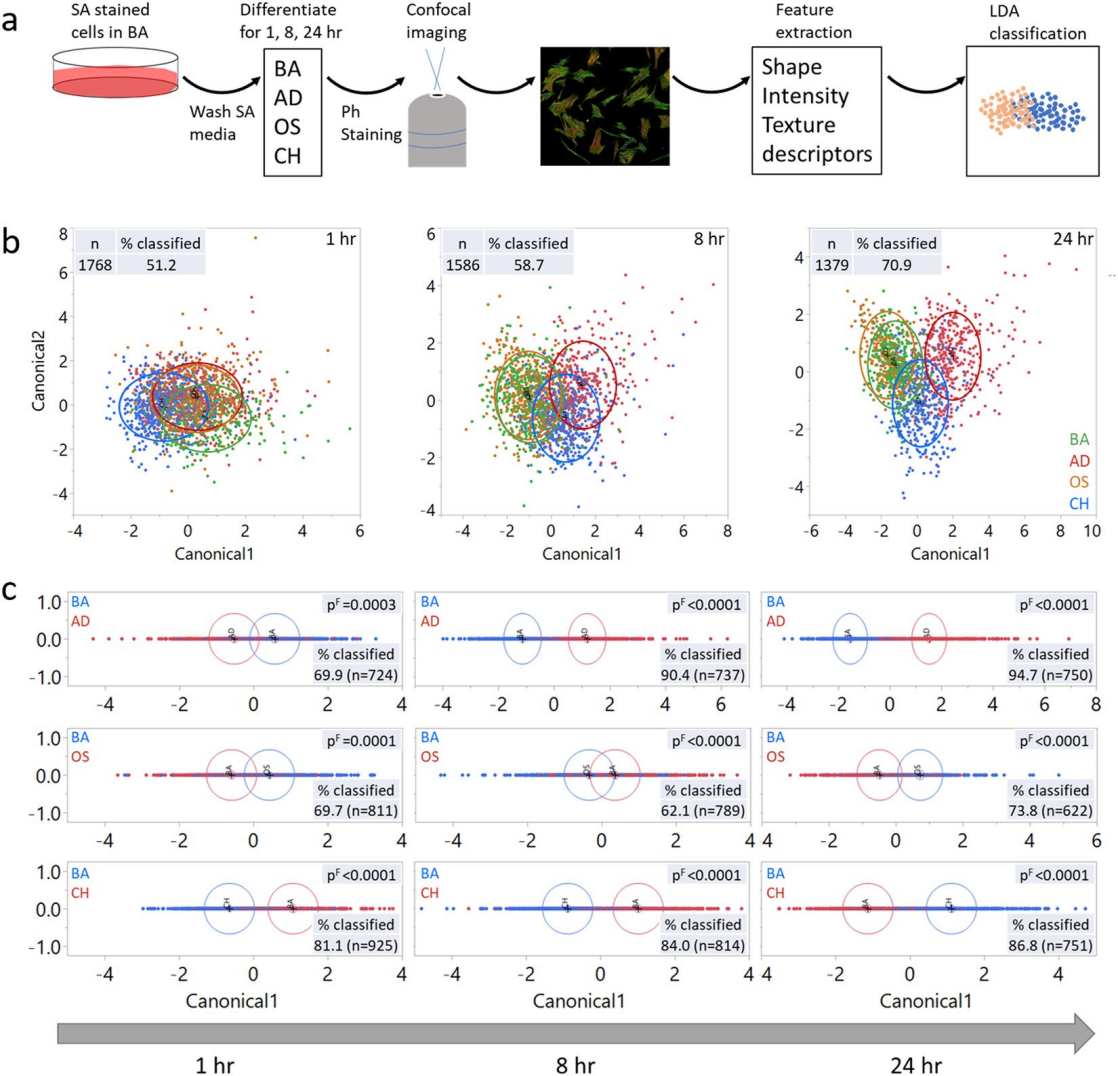
F-actin dual staining coupled with high-content analysis for parsing MSC differentiation. (**a**) Workflow describing the high-content image analysis. SA stained cells were cultured in AD, OS or CH media for 1, 8, or 24 hrs. The cells were fixed and stained with a competing F-actin stain, phalloidin (Ph) and images were acquired using confocal microscopy. A total of 41 shape, intensity and texture-based features were extracted from single cells. These features or descriptors were used as the input to derive canonical covariates using linear discriminant analysis (LDA) to classify the individual cells. (**b**) Simultaneous comparison of cells cultured in AD, OS and CH media to show early divergence in the cytoskeleton during differentiation. Each point represents a cell. Contour plot encompasses roughly 50% of the cells in a given media, while its center approximates the mean canonical values. (**c**) Binary classification of cells highlight temporal divergence of cytoskeleton during differentiation. Wilks’ lambda test was employed to calculate the p-value, p^F^ to compare the means of the covariates among the groups.

SA staining of single stem cells also enables higher content analysis of the temporal progression of the cytoskeleton morphology during differentiation. A single cell morphometric approach was employed to quantitatively profile cytoskeletal changes during the early lineage divergence. The workflow as described in Fig. 4a involved differentiation induction of SA-stained cells, followed by complementary Phalloidin staining and confocal imaging. Single cell images were analyzed and high content image informatics (HCIA) was performed to allow classification of single cells in BA, AD, OS and CH groups using linear discriminant analysis (LDA) (Fig. 4a). Each point on the plot represents the data from a single cell, the contour plots envelope roughly 50% of the cell population, and the center of the contours approximates the mean values of the canonical variables (Fig. 4b,c). Examination of tri-lineage progression showed temporal divergence in the cytoskeleton (Fig. 4b). After 1 h of induction, all medias had largely overlapping contour plots, and the number of correctly classified cells was 51.2%. The data from 8 h timepoint demonstrated increased separation of the contours with improved classification of 58.7%. After 24 h, the contour plots were more separated, as evidenced by improved classification of 70.9%. These results demonstrate that the early stage cytoskeletal changes during MSC differentiation can discern lineages within 24 h in a lineage specific manner. In the interest of decluttering the data, each lineage was also individually compared with the basal condition (Fig. 4c). 70–80% of the cells were correctly classified with significant differences in the mean values of the covariates as early as 1 h. At 8 h, the cells clustered more distinctly except in the OS media. At 24 h, ~95%, 74%, 87% of the cells were classified in separate lineage groups, AD, OS and CH, respectively. Note that the difference in the mean canonical values were significant in all conditions at all timepoints, while the single cell classification was a function of time. The high-content image analysis demonstrated that MSC differentiation involves temporal morphometric changes in the actin cytoskeleton, which are evident as early as one-hour post-induction.

### Quantifying altered actin dynamics in MSCs following aging in culture

**Figure 5:**
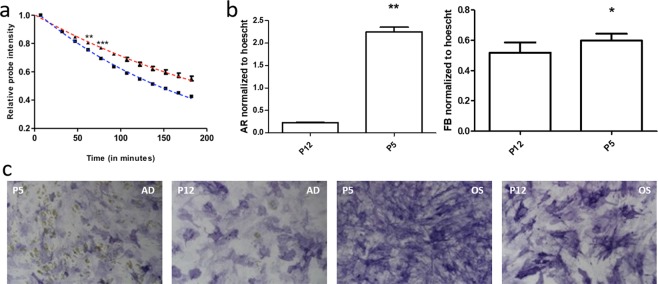
Assessment of altered actin turnover kinetics and differentiation due to *in-vitro* aging. (**a**) SMAT analysis discerned early passage cells (P5, blue curve) from late passage (P12, red curve) cells. Values are ratiometric mean ± standard error of SA intensity normalized to the first timepoint within the respective group. **p < 0.01, ***p < 0.001 vs the P5 group (n = 3). (**b**) Quantification of Fast Blue (FB) and Adipored (AR) from B. For each group, values are mean + standard error of AR or FB normalized to Hoechst staining. *p < 0.05, **p < 0.01 vs the P5 group (n = 3). (**c**) Late passage (P12) cells showed minimal lipid droplet accumulation after adipogenic induction (AD) and reduced fast blue staining after osteogenic induction (OS) compared to early passage cells (P12).

Cellular aging is a complex phenomenon which is associated with reduced telomere length, altered transcriptional profile, DNA damage and epigenetic deregulation^18^. Aging in MSCs leads to a reduction in the proliferative capacity and differentiation potential. Since we were able to detect subtle changes in actin turnover in response to the lineage specific cues, we hypothesized that SMAT could be harnessed to detect changes in the actin turnover due to *in vitro* aging. The effect of *in vitro* cellular aging on actin turnover was evaluated by simultaneous SMAT profiling with early (P5) and late (P12) passage MSCs. P12 cells exhibited slower SA decay compared to the P5 cells, and the data reached statistical significance after 1 h (p < 0.01 at 62 min, p < 0.001 at 77 min after SiR-actin removal) (Fig. 5a). In a parallel study, P5 and P12 cells were evaluated for adipogenesis and osteogenesis for 14 days. P12 cells showed significantly reduced adipocytes (cells with lipid droplet accumulation) and osteoblasts (fast blue stained cells) compared to P5 cells (Fig. 5b,c). Therefore, SA decay could be used as a marker to evaluate and forecast diminished differentiation potential in MSCs due to *in vitro* senescence.

## Discussion

The actin cytoskeleton plays a pivotal role in guiding MSC differentiation^4,6^. The mechanobiology, signaling pathways, and morphology of the actin cytoskeleton have been extensively studied in the context of MSC differentiation^4,19–22^ but the kinetics of the actin reorganization are mostly unexplored. We reasoned that change in cell shape begins by a highly organized and complex restructuring of the network of actin filaments. Given that actin reorganization is accompanied by a change in actin turnover^16,23,24^, the actin turnover has the potential to be a real-time indicator of the inherent cytoskeletal dynamics. In this study we developed a novel approach to monitor the actin turnover and parsed the kinetics of actin turnover during chemically induced MSC differentiation and *in-vitro* senescence. The key reagent for the proposed method is a fluorogenic probe, SA, which binds select F-actin sites depending on *in situ* cellular dynamic states^15^.

Initial validation of SA based quantification of changing actin turnover was conducted with the cytoskeletal drugs that are known to perturb the actin cytoskeleton. NOC was used as a negative control as it induces depolymerization of microtubules without interfering with actin assembly^25–27^. At high dose (8 uM), NOC resulted in reduced cell size, but had no effect on SA staining. Treatment with actin perturbing drugs showed more dramatic effects on SA staining. CYTO binds the plus end of F-actin, thereby interferes with addition of G-actin leading to arrested actin dynamics^28–30^. At high dose (1 uM), CYTO caused the cytoskeleton to collapse, whereas SA was retained due to encumbered actin turnover. In contrast, high dose (1 uM) of JASP caused rapid decline in SA staining by promoting de novo filament formation and possibly due to the structural similarity with the probe^31^. Treatment with low drug dose showed that SA staining is capable of detecting actin cytoskeletal perturbations even without any observed change in cell shape. Furthermore, we also investigated the influence of serum deprived media (SDM) on SA staining. We were able to detect changes in actin turnover during both serum reintroduction (SDM- > BA) and initiation of serum deprivation in the complete media (BA- > SDM) (Fig. 2c) as early as 15 minutes.

The primary goal of this study was to discern possible temporal changes in actin turnover during MSC differentiation. Using imaging of SA actin-based turnover (abbreviated as SMAT) and dual-staining based HCIA, we report altered actin dynamics and organization much earlier than seen using conventional methods. The earliest changes in the cytoskeleton during differentiation have been reported based on mechanobiology (Change in young’s moduli at day 10^32^), morphometric descriptors (after 1 day^9^) and phalloidin intensity (after 1 day; flow cytometry^10^). With SMAT, we discerned changes in actin turnover as early as few minutes upon the induction of lineage differentiation. To establish that the observed change in actin turnover is related to the lineage commitment, we employed a converse approach by inducing dedifferentiation. Within minutes of a differentiation media swap (by removing lineage differentiation factors), we observed a switch in the SA decay rate, as lineage committed cells assumed decay profiles of cells in basal media, which subsequently led to reduction in the number of adipocytes compared to the group with no media change (Fig. 3d,e). The media swap study provided evidence for high responsiveness and plasticity of the actin cytoskeleton. Therefore, SMAT has the potential to be used as a robust early indicator for actin turnover, and the tested cells can be used for subsequent assays to verify the functionality. As a potential application, we showed a correlation between a down-tick in actin turnover with MSC differentiation due to *in-vitro* aging (Fig. 5).

Using the SMAT profiling, we could effectively discern between AD, OS and CH lineages, but the differences between BA and OS remained insignificant. This could be potentially due to similar actin dynamics of BA and OS during the time course study. Previously, we employed high content informatics to parse BA from OS by 24 hours but on fibronectin coated surfaces that are conducive to osteogenesis. While, with uncoated surface, it took 48 hours to discern the two^9^. The analogous approach to our 2009 paper in this study is the s-p dual staining based descriptor analysis where we could extract an array of descriptors that enabled us to correctly classify osteogenic cells with ~70% accuracy as early as one hour of induction (Fig. 4c). Due to dual chromatics of phalloidin and SiR-actin, we generated more richer descriptors that enabled earlier classification. Simultaneous staining with complementary probes, SA and Ph, provides a new strategy by which the F-actin sites can be labeled based on the inherent turnover dynamics. After removing the SA media, the residual SA stain represents the F-actin sites that were not turned over. Subsequent staining with Ph will specifically highlight the unoccupied F-actin sites. We also confirmed the competitive nature of the probes in a dose response study (Supplementary Fig. 3b). The dual-actin image informatics demonstrated the temporal drift of single cells during lineage progression that eventually was able to cluster cells in all 3 lineages (AD, OS, and CH) simultaneously after 24 hours of induction. A binary juxtaposition of each lineage with the basal condition improved the correct classification, and also displayed the actin structural divergence that accompanies lineage progression. The features that enabled the classification of cells were identified by a predictor screening on JMP to rank the features. SA intensity and haralick features were the top 5 predictors across all conditions (Supplementary Table 2), while notably the shape descriptors did not feature as the influential ones. Therefore, high content image informatics enabled identification of phenotypic variations in the cytoskeleton in advance of the role of the cell shape. High content image informatics-based parsing improved with time, indicating that the cytoskeleton undergoes progressive reorganization during lineage commitment. Unlike SMAT which is based on a unidimensional analysis, high content image informatics benefits from multi-variate approach that enables better classification. However, the two major limitations of high content informatics are that these are based on discrete timepoints and require complex data analysis. On the other hand, SMAT is suitable for dynamic profiling of actin turnover over prolonged durations.

**Figure 6:**
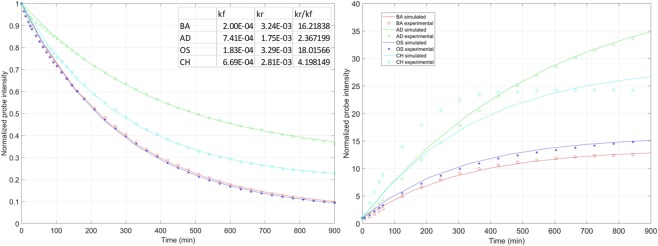
The lineage-specific divergence of normalized SA kinetics was modeled in live MSCs. Kinetic data for SA decay (Fig. 3a), and SA association (Fig. 3b), were plotted in the LEFT and RIGHT panels, respectively, and denoted using varied symbols for different treatment conditions above (BA: basal; AD: adipogenic; OS: osteogenic; CH: chondrogenic). Kinetic parameters were estimated using a simple ligand-receptor binding model to fit the data and used to generate simulated kinetics (bold curves). The table in the left panel shows the values of the two rate constants, kr(dissociation) and kf (association). The ratio kr/kf indicates lineage specific dissociation kinetics for SA. (See materials and methods for details).

In an attempt to explain the observed SA kinetics during lineage progression, we proposed a simple ligand-receptor equilibrium model simulating the observed data (Fig. 6). First, the kinetic parameters were fitted to the SA dissociation kinetics in Fig. 3a, and then used to determine the SA association in Fig. 3b (Fig. 6), showing that both association and dissociation can be adequately described by a consistent set of kinetics parameters. The proposed model supports a ligand-receptor interaction model of SA with F-actin (Fig. 6). After removal of SA from the growth media, the initial fast decay is likely the result of rapid loss of SA primarily from the dynamic structures within the actin cytoskeleton, such as podosomes and filopodia. On the contrary, stress fibers show SA staining even after several hours. We suggest that the least dynamic regions are primarily responsible for lineage specific changes in the turnover, as they are the prime sites responsible for the change in SA. The SMAT analysis of early changes during adipogenic and chondrogenic differentiation show a major decrease in actin turnover, whereas osteogenic differentiation is accompanied with a rapid actin turnover. This observation is supported by the previous findings where osteogenic differentiation leads to extensive reorganization of actin filaments to create a disordered mesh with thick stress fibers^6^. In contrast, adipogenic differentiation showed disrupted network, which may be the result of arrested actin dynamics^4,33^. The proposed model suggests the need in the future for a more detailed biophysical analysis of the interaction of SA with F-actin during MSC lineage progression, and the overall role of actin cytoskeleton during immediate early stages of MSC differentiation.

MSCs possess the ability to proliferate and differentiate, but because of their low frequency, it is necessary to expand them *in vitro* for clinical applications. During *in vitro* expansion, these cells are susceptible to a decline in the proliferative capacity along with reduced differentiation potential^34^. Given that increasing passage number leads to an altered cell morphology we hypothesized that SA label could be used as a dynamic function for evaluation of change in actin turnover due to *in vitro* aging^11^. Using SMAT, we could detect altered actin turnover in late passage cells within one hour of the assay. Thus, SA decay-based evaluation of actin turnover could be used as a potential tool for benchmarking the actin turnover to evaluate *in vitro* aged MSCs or from aged donors to predict their differentiation potential.

The influence of SA on MSC morphology and differentiation was also examined (Supplementary Figs 2, 3). Complex structures within the actin cytoskeleton with slow actin turnover (such as stress fibers) showed bright staining unlike the dynamic regions towards cellular periphery that are involved in motility (Supplementary Fig. 3a). Due to structural similarity with JASP, SA has the potential to influence actin dynamics and morphology^15^. We compared the area of SA stained and unstained cells after fixing and independent phalloidin staining (supplementary Fig. 3C). At 100 nM, SA treated cells displayed increased cell area but the difference was not significant. However, a higher SA dose (500 nM) resulted in distinct change of cell shape from elongated spindle to a rounded morphology along with more pronounced stress fibers in comparison with the low SA dose (100 nM) (Supplementary Fig. 3a,c). Therefore, low SA dose was found to be suitable for the live imaging of MSCs. Thus, SMAT has the potential to assess actin turnover over the course of several hours with a simple low magnification imaging protocol. Further, SMAT analysis may be suitable for bulk cell populations where single cell segmentation analysis may be challenging. Most of the conventional methods for *in vitro* MSC quality control, such as analyzing the gene expression^35^, differentiation^36^, immunostaining^37^ and senescence^38^, are difficult to implement with live MSCs, and thus cannot be used in preparative processing of cells prior to therapy. At low dose (100 nM), transient labeling with SA, is suitable for live characterization of MSC behaviors without adversely affecting the innate MSC ability to proliferate or differentiate (Supplementary Figs 2, 4). We note the caveat that at higher concentrations, SA staining could induce cytoskeletal reorganization, and as such, the effect of labeling should be independently investigated to determine its influence on cytoskeletal dynamics and other cellular functions. Additionally, supplementation of 100 nM SA with verapamil, a broad-spectrum efflux pump inhibitor recommended by the SA manufacturer to improve signal, caused prolonged retention of the probe resulting in poor or delayed resolution of decay plots, and hence was not used in this study.

Most of the information about actin dynamics in live cells has come from assays based on FRAP (fluorescence recovery after photobleaching) based experiments [49]. The recovery of fluorescence is driven by intrinsic diffusion kinetics of depolymerization of bleached monomers and addition of new non-bleached monomers on the filaments^39^ [50]. On the other hand, the SMAT approach is primarily driven by SA dissociation from F-actin sites during depolymerization and a slow association rate of the probe, leading to a net temporal decline in number of labeled actin sites^14^. While both methods measure actin turnover, these approaches could offer complementary information about the actin dynamics. FRAP is well characterized for monitoring specific region of interest within a single cell for short duration (up to few minutes), while SMAT could be used to monitor the global actin dynamics of cell populations for long-term (several hours). In addition, due to the low magnification imaging protocol and ease of data analysis, SMAT could be readily applied for high-throughput analysis of multiple cell populations.

This study revealed the early changes in the kinetics and organization of actin turnover in response to differentiation induction and *in vitro* aging. The earliest reported change during adipogenic differentiation involved altered expression of 46 genes as early as 30 minutes^40^. Subsequent analysis in the same paper and other studies have also demonstrated upregulation of adipogenic transcription factors in the C/EBP family and MYC^41,42^. Notably, using imaging of the SA turnover, we observed an even earlier decrease in actin turnover during cell culture in the adipogenic media. This raises the question about the causality i.e. whether the change in actin dynamics is a result of altered transcriptional profile or whether the actin dynamics initiate MSC differentiation via downstream signaling. In the latter scenario, actin might influence the immediate-early gene expression with integrin, Rho and YAP/TAZ signaling pathways^37,43^. Actin monomers have been shown to be a part of the transcriptional apparatus that modulate gene expression^44^. Also, the proportion of G to F actin determines the activity of a transcription cofactor, serum response factor (SRF) along with its cofactor MKL1^45^, which have been shown to be an important regulator of MSC differentiation. On the other hand, if the actin-transforming signal emanates from the nucleus then the focus would be on immediate early genes that have been reported to be associated with both MSC differentiation and the cytoskeleton. Our previous gene expression analysis shows several clusters of genes upregulated early during lineage divergence, including cell cycle genes and nuclear splicing factor genes, especially Nuclear mitotic apparatus protein, NuMA^46^. Morphotextural descriptors of NuMa were able to distinguish phenotypes with greater efficacy when combined with descriptors for actin filaments in MSCs^47^.

In summary, we have described an approach to study actin turnover and organizational features using the probe, SA. First, we validated SA dissociation turnover dynamics by quantifying the early effects of cytoskeletal perturbation on actin turnover. Next, we found that MSC differentiation involves change in actin turnover that precedes change in the cell shape and has the potential to be used as a dynamic reporter of lineage progression. A single-cell F-actin dual-staining based high content image informatics approach enabled parsing emergent phenotypes much earlier than the previously reported studies. Finally, as a potential application, we applied the SA turnover image analysis to detect slow actin turnover as a result of *in-vitro* aging, which could be a potential discriminant of early aging effects in stem cell cultures.

## Materials and Methods

### Mathematical model for SA decay kinetics

SA binding with F-actin sites is dependent on the actin dynamics. The observed change in intensity of the SA probe in the time-lapse images can be illustrated using a ligand-receptor binding model, where the unbound probe (ligand, L) associates with the F-actin site (Receptor, R) resulting in 100-fold increase in fluorescence of the probe (C) in a reversible manner.

$$L+R\leftrightarrow C$$

*k*_*f*_ and *k*_r_ represent the rate constants for forward and backward reaction. C_0_ is the initial total fluorescent probe bound with F-actin. Due to the inherent actin dynamics, there is a net dissociation of SA probe as it comes off from F-actin and loses fluorescence by 100 fold. But, the rate constants *k*_*f*_ and *k*_r_ dependent on the lineage progression towards AD, OS or CH. Hence, we observed different lineage specific decay rates. The decay kinetics in Fig. 3a can be described as follows:

$$\frac{dC}{dt}={k}_{f}{R}_{T}({C}_{0}-C)-{k}_{r}C$$

Where *C*_0_ − *C* denotes the concentration of free ligands while the available binding sites are denoted by *R*_*T*_ and are assumed to be in excess. Fitting the dissociation kinetics enabled us to deconvolute the values of the parameters: ${k}_{r}\,{\rm{a}}{\rm{n}}{\rm{d}}\,{k}_{f}^{d}={k}_{f}{R}_{T}$

On the other hand, Fig. 3b describes a scenario where the net effect is the association of SA resulting in increase in the intensity with time. Now, we assume L_T_, the total number of probe molecules, to be constant. The association kinetics, assuming an excess on unbound ligands (*L*_*T*_), in Fig. 3b can be described as follows:

$$\frac{dC}{dt}={k}_{f}{R}_{T}{L}_{T}-{k}_{r}C$$

The effective rate of association, ${k}_{f}^{a}={k}_{f}^{d}{L}_{T}$, depends on the media to reduce actin turnover. Therefore, using the association data we can estimate *L*_*T*_. The SA association described in Fig. 3b initiates with the abundant availability of the SA ligand, which results in rapid increase in C.

The model parameters were successively estimated: using the dissociation data we determined ${k}_{f}^{d}\,{\rm{a}}{\rm{n}}{\rm{d}}\,{k}_{r}$ and subsequently using the association data we determined *L*_*T*_. Figure 6 shows the plots of estimated and the experimental data, except CH in the association study, the estimated plots corroborated with the observed data. The ratio of kr/kf could be used as a parameter to describe the simulated dissociation kinetics of SA at equilibrium.

### Cell culture

Human bone-marrow derived MSCs were a kind gift from Dr. Rick Cohen (BME, Rutgers University). Cells were cultured in Corning® T-75 tissue culture flasks in humidity-controlled 5% CO_2_ atmosphere at 37 °C. After the initial expansion of the cells in the peprotech media, cells were maintained in the basal growth media (BA) composed of Gibco Minimum essential medium α (MEMα) supplemented with 10% premium-select fetal bovine serum (Atlanta Biologicals), and penicillin-streptomycin 0.1% v/v (Lonza). Cells were received at passage 3 and were used till passage 8 unless specified otherwise. The growth media was changed every third day and cells were passaged when the monolayer reached 70–80% confluence. For dissociating the cells, TrypLE™ express enzyme (Gibco) was used and seeding density for maintenance was typically 3,000–4000 cells per cm^2^. Adipogenic media (AD) was prepared by supplementing BA with final concentrations of 1 μM dexamethasone, 10μg/mL insulin, 500 μM isobutyl-1-methyl-xanthine and 200 μM indomethacine. Osteogenic media (OS) were prepared by supplementing BA with 500 μM L-ascorbic acid-2phosphate, 1 μM dexamethasone, and 10 nM b-glycerophosphate. Chondrogenic media bullet kit was obtained from Lonza. For inducing differentiation, cells were passaged to 96-well dish, allowed to attach overnight and induction media was added next day and subsequently changed every third day. The cytoskeletal drugs, jasplakinolide and cytochalasin D were purchased from Cayman chemicals, and nocodazole from Sigma Aldrich.

### Live cell staining with SA and imaging

MSCs were seeded on 96 well tissue culture treated imaging dishes (Corning) and allowed to attach for 2 hrs. Then 100 nM SiR-actin (Cytoskeleton, Inc.) was added to the BA media for overnight staining of live cells. For time-lapse imaging, Zeiss LSM780 laser scanning confocal microscope was used. The tissue culture dish was placed on the microscope equipped with a stage-top incubator and staining media was aspirated, followed by quick media change to remove residual SiR-actin. Immediately, the first image-set was taken for all the test conditions as base intensity reference picture. The cells were then treated based on the experimental setup and time-lapse imaging was performed to capture the same fields at multiple time points. Imaging was done with the 10x objective in triplicates for all test conditions and 4 distinct fields were imaged in each replicate.

### Measurement of intensity and data analysis

After image acquisition, the intensity of SiR-actin was quantified by measuring mean gray value on ImageJ. The mean gray value for all the image sets for a specific field of view were normalized to the first timepoint, and the values were averaged by the number of replicates and fields of view for each condition. The averaged mean gray value was plotted with time to quantify actin dynamics using SMAT.

### F-actin dual staining based high content image analysis

Following prolonged SA staining in BA, the cells were stimulated with AD, OS or CH media and stained with phalloidin-488 (Ph). 10 × 10 Tile scans of single cells were generated with the 20x objective with the confocal microscope. The images were analyzed on CellProfiler, and single cells were identified based on Hoescht as reference; while the cytoskeletal segmentation was performed based on Otsu thresholding. 41 morphometric features describing the cell shape, intensity of the fluorophores (s-p) and texture (Haralick features) of the cytoskeleton were obtained for each cell. The descriptors are listed in Supplementary Table 1. Linear discriminant analysis (LDA) was performed on JMP software for dimensional reduction to categorize the differentiating cells. Briefly, linear combination of 41 variables were used to derive canonical variables that represent the variations among BA, AD, OS and CH groups (Supplementary Table 1). The percent of correctly classified cells was calculated based on the specified data set. In the canonical plot, an ellipse that contains 50% of the data sets were drawn where the center represents the 95% confidence region for the means of the canonical variables. Wilk’s Lambda test was performed to compare the means of the covariates (41 features) across groups. In addition, the Predictor screening platform on JMP was utilized to identify the significant predictors of differentiating cells based on bootstrap forest model.

### Phalloidin staining

MSCs were fixed with 4% paraformaldehyde (Electron Microscopy Sciences) for 15 minutes, permeabilized with 0.1% Triton X-100 (Sigma) in PBS. To visualize F-actin, Alexa Fluor™ 488 Phalloidin (Life technologies) was used after dilution in PBS.

### Cell differentiation assay

After 14 days of induction with differentiation media, the cells were fixed and stained with fast blue RR (Sigma) and AdipoRed (Lonza) reagents to stain for alkaline phosphatase (osteoblast) and intracellular triglycerides (adipocyte). AdipoRed and fast blue were quantified on the Tecan microplate reader by measuring fluorescence with excitation at 485 and emission at 535 nm. Fast blue absorbance was measured at 572 nm.

### Cell proliferation assays

The CellTiter 96® AQueous One Solution Cell Proliferation Assay (Promega) was performed to assess the effect of SA staining on cell viability. Briefly, cells were cultured at a density of 10,000 cells/cm^2^ in a 96 well plate and labeled with 100 nM SA in either complete basal media (BA) or serum deprived media (SDM) for 18 hours. After overnight staining, the media was replaced with fresh media and the assay was performed along with unstained cells after 24 or 48 hours of initiation of SA labeling. To assess cell viability, Aqueous One Solution was added in each well to be assayed and incubated for 1 hour. The absorbance was measured using a plate reader at 490 nm.

### Immunofluorescence analysis of ki67

The expression of ki67 in MSCs was quantified by immunofluorescence. Cells were cultured at a density of 3,000 cells/cm2 in 96 well plate and labeled with 100 nM SA in either complete basal media (BA) or serum deprived media (SDM) for 18 hours. After overnight staining, the media was replaced with fresh media and the cells were fixed with 4% paraformaldehyde in PBS for 10 minutes after 24 or 48 hours of initiation of SA labeling. Fixed cells were incubated in the blocking buffer (10% normal goat serum, 1% BSA, 0.1% Triton-X100 in PBS) for one hour at room temperature. Cells were incubated in the primary antibody for ki67 (Abcam:15580) overnight at 1:200 dilution in 4 degrees, followed by three 5 minute washes. Subsequent, secondary antibody (Invitrogen, A-11010) staining was done at dilution of 1:500 for 1 hour at room temperature. After three 5 minute washes, nuclei was stained with Hoescht in PBS for 5 minutes. For quantification of ki67 expression in single cells, confocal imaging was performed. Nuclei was used to designate ROI for ki67, intensity was measured with ImageJ.

### Statistical analysis

Statistical analysis for SMAT data was done with GraphPad 5 software. The analysis of the changing probe intensity from live-imaging was performed by two-way ANOVA followed by Bonferroni post-hoc correction of p-values. While, with the fixed cells, one way repeated measures ANOVA was done followed by Turkey’s multiple comparison test. Asterisks were used to show the significance with the following p-values: p < 0.05,**p < 0.01, ***p < 0.001. Statistical analysis for LDA was done on JMP software as described above using the Wilks’ Lambda test to compare the means of the covariates (41 features) across groups (BA, AD, OS and CH) based on the approximate p-values, p^F^.

## Supplementary information

Supplementary Information

Supplementary Video 1

Supplementary Video 2

## Data Availability

The data-sets generated during and/or analyzed in this study are available from the corresponding author upon request.
