# Supplementary material for: Fluorescence Imaging of Actin Turnover Parses Early Stem Cell Lineage Divergence and Senescence
Source: Sci Rep. 2019 Jul 17;9:10377. doi: 10.1038/s41598-019-46682-y (PMC6637207; doi:10.1038/s41598-019-46682-y)
Supplement: Supplementary file 1 — Supplementary Information [file 41598_2019_46682_MOESM1_ESM.docx]

**SREP-19-06435A**

**Fluorescence Imaging of Actin Turnover Parses Early Stem Cell Lineage Divergence and Senescence**

Prakhar Mishra, Daniel C. Martin, Ioannis P. Androulakis and Prabhas V. Moghe*

**
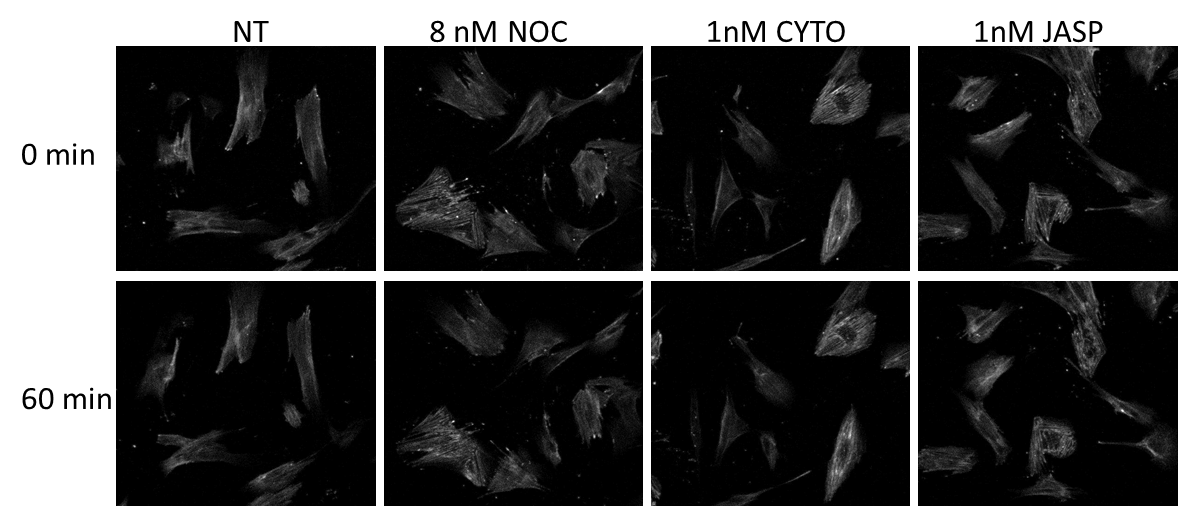
**

**Supplementary Fig 1: Cytoskeletal drug treatment at low dosage had no appreciable effect on cell morphology.** SA stained cells were treated with the cytoskeletal drugs NOC, CYTO and JASP after washing off the staining media and imaged for 60 minutes.

**Supplementary Fig 2: Effect of SA staining on MSC differentiation.** Cells were either stained once (day 0) or twice (day 0 and 7) overnight during 14 days of AD or OS differentiation. Adipogenesis (adipored) and osteogenesis (fast blue) were compared vs unstained group. For each group, values are mean +standard error (n=3).


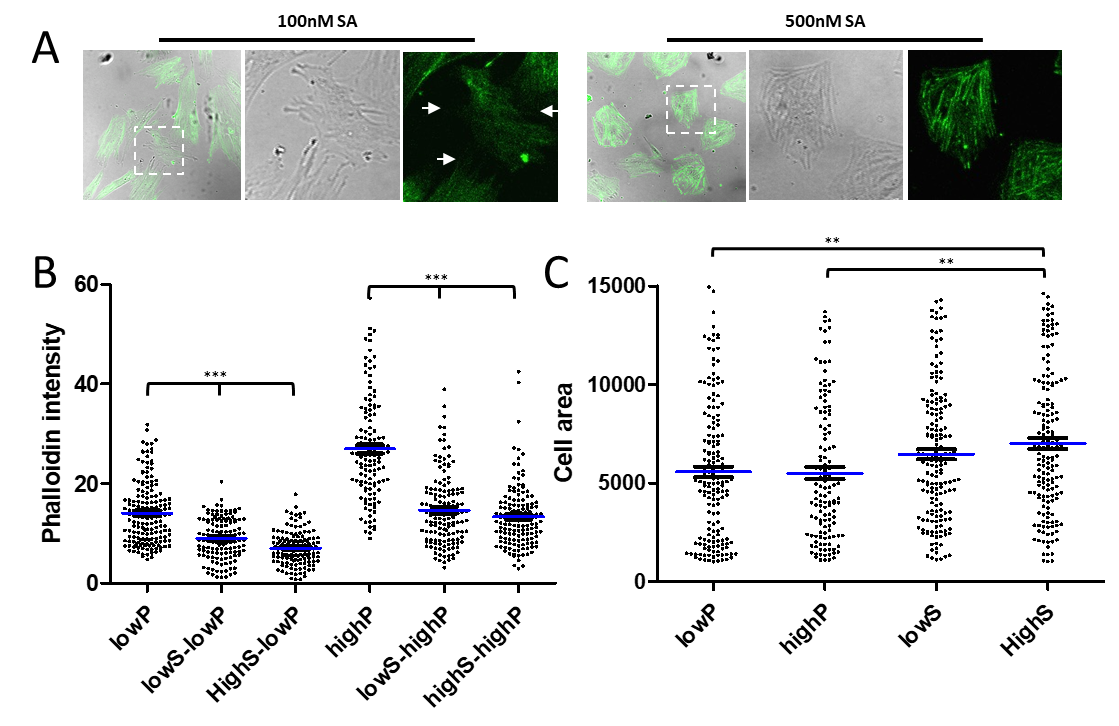


**S****upplementary Fig 3:** **Characterization of the effect of SA on actin morphology and co-staining with Phalloidin**

**(A)** Live cell images after staining with SA (green) at 100nM and 500nM. 100nM SA showed poor staining of protrusive dynamic structures such as filopodia (white arrows) but brighter staining of stress fibers. The dashed rectangle is the zoomed-in region to show single cells. 500nM SA stained cells affected the cell morphology accompanied by increase in stress fibers. **(B)** Phalloidin (P) staining is inversely correlated with SA. Cells stained with high(500nM) or low (100nM) SA were subsequently stained with high (132nM) or low (26.4nM) concentration of P. The mean intensity of P + standard error shown in blue bars. ***p<0.001 vs the single stained group (n=130-170) **(C)** SA labeling led to increase in cell area, but the differences were not significant at 100nM. For each group, mean area± standard error is shown in blue bars. **p<0.01 was calculated within groups with Turkey’s multiple comparison test (n=130-170)

**
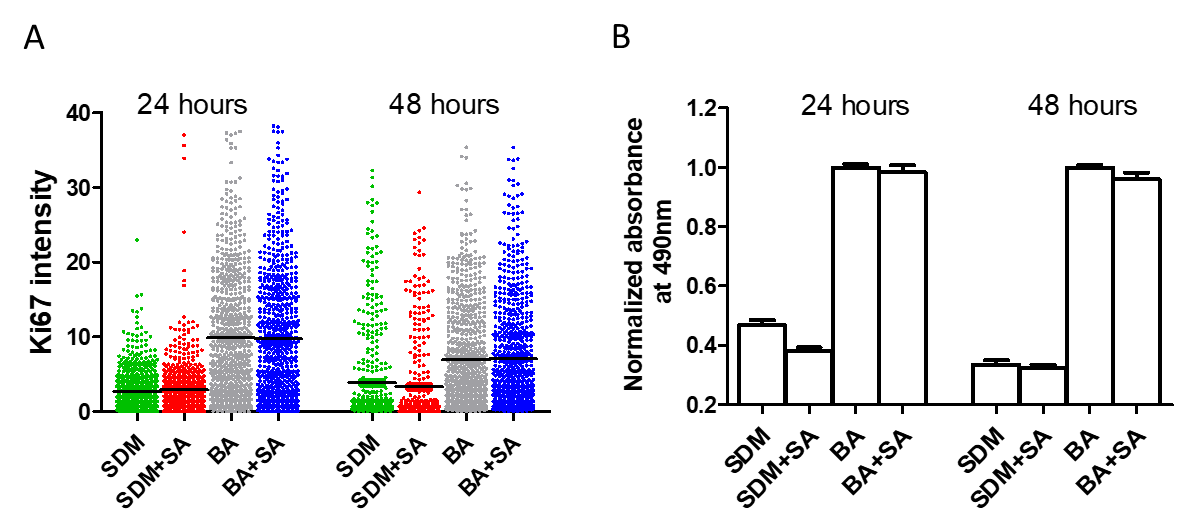
**

**Supplementary Fig 4: Effect of SA staining on cell proliferation**

Cells were stained with SA for 18 hours in either basal media (BA+SA) or serum deprived media (SDM+SA). Cell proliferation and viability were assessed by comparing **(A)** ki67 expression and **(B)** MTS assay respectively, with unstained cells (BA or SDM) after 24 and 48 hours of initial staining. Cells in serum deprived media (SDM) were used as negative control. No significant differences were observed in either assays due to SA labeling in either media. Mean Ki67 intensity (black bars) was higher in both BA and BA+SA as determined by one-way ANOVA (p<0.001 vs SDM or SDM+SA, n=400-742 cells). Similar results were obtained with MTS assay (p<0.001 vs SDM or SDM+SA, n=5)


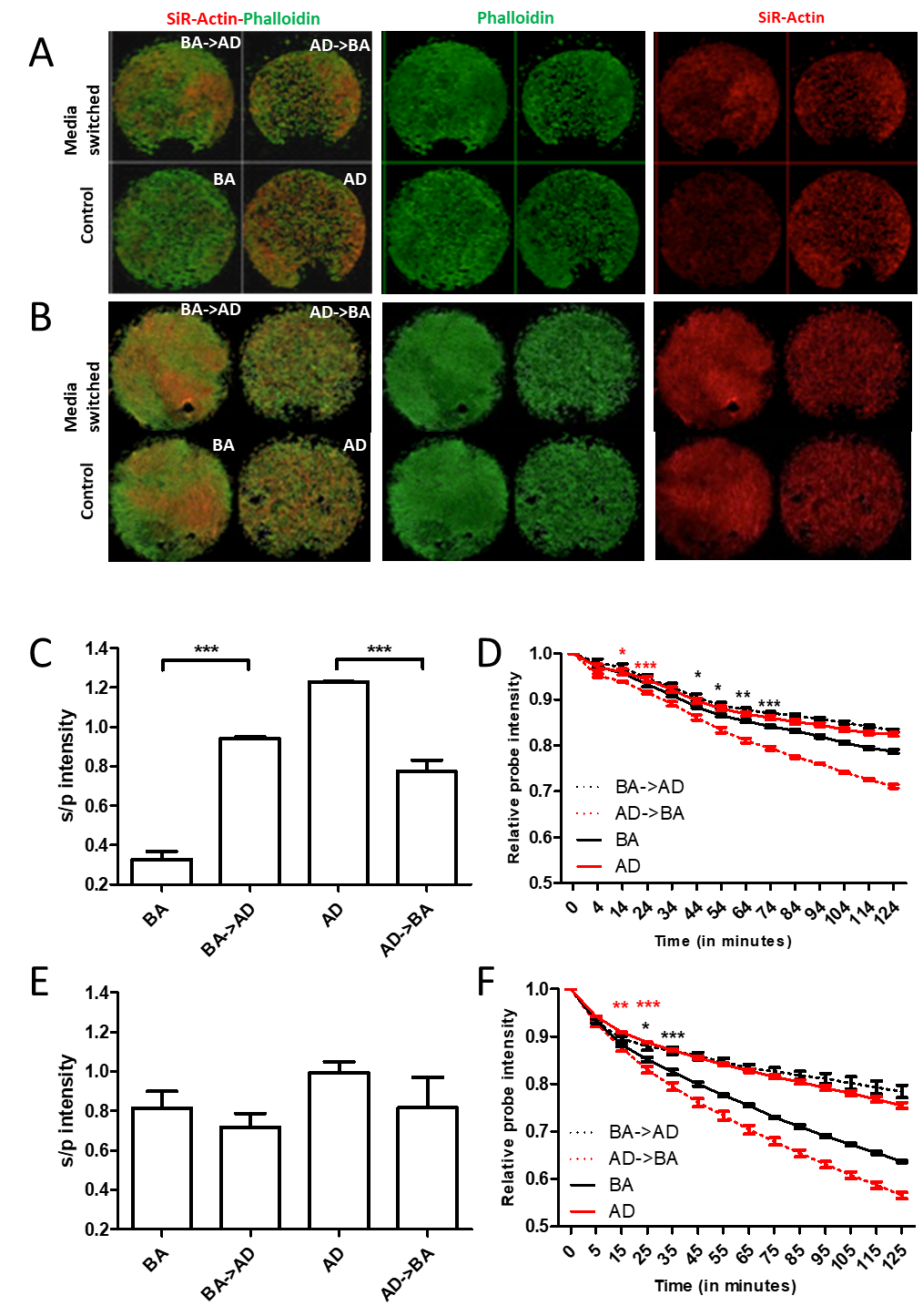


**Supplementary Fig 5. SA vs. Phalloidin (s/p) staining demonstrates cytoskeletal plasticity after media switch during MSC differentiation.** Cells were cultured in basal (BA) or adipogenic media (AD) for 3 or 14 days prior to media switch. A night before the media switch, cells were stained with SA and after 3 hours of media switch cells were fixed and stained with phalloidin.

A, B. s-p dual stained cells after 3 days (A) or 14 days of induction (B)

C, E. S/P intensity ratio for figures A and B respectively. For each group, values are mean +standard error (n=3). ***p<0.001 within groups with Turkey’s multiple comparison test
D, F. SMAT analysis after media switch at 3 days (C), and 14 days (F) post-differentiation induction. For each group, values are ratiometric mean ± standard error of SA intensity normalized to the first timepoint within the respective group. *p<0.05, **p<0.01, ***p<0.001 vs the BA group (n=3).

| Category | Feature | Canon1 | Canon2 | Canon3 |
| --- | --- | --- | --- | --- |
| Shape | Area | -0.0921 | 0.638692 | 0.07808 |
|  | Compactness | 0.15783 | 0.194831 | 0.243956 |
|  | Major Axis Length | -0.3228 | 0.231322 | -0.68679 |
|  | Max Feret Diameter | 0.13517 | -0.82015 | 0.766212 |
|  | Min Feret Diameter | 0.03169 | -1.0574 | 0.975473 |
|  | Minor Axis Length | -0.2893 | 0.964815 | -0.90758 |
|  | Perimeter | 0.11993 | 0.517484 | -0.26398 |
|  | Solidity | -0.0592 | 0.171664 | 0.230162 |
| Intensity | Manders_Correlation | -0.0014 | 0.038961 | 0.185267 |
|  | Integrated Intensity_ph | 0.13481 | -0.47987 | 0.825335 |
|  | Integrated Intensity_sir | -0.3033 | -0.58467 | -0.51118 |
|  | Mean Intensity_ph | -0.5487 | 0.161539 | -0.33304 |
|  | Mean Intensity_sir | 1.23095 | 0.556185 | 0.286562 |
| Haralick texture features | Angular Second Moment_ph | -0.1563 | -0.89925 | -1.60342 |
|  | Angular Second Moment_sir | -1.016 | -0.79401 | 3.917558 |
|  | Contrast_ph | 0.2882 | 0.856925 | 0.295049 |
|  | Contrast_sir | 1.41222 | -0.3294 | -3.03077 |
|  | Correlation_ph | 0.39667 | 0.348032 | -0.15778 |
|  | Correlation_sir | 0.22622 | -0.30291 | 0.718908 |
|  | Difference Entropy_ph | -0.115 | -0.17024 | 1.3306 |
|  | Difference Entropy_sir | 1.61033 | -1.57163 | -2.69159 |
|  | Difference Variance_ph | 0.22155 | -0.17868 | -0.73826 |
|  | Difference Variance_sir | -0.9047 | 0.872723 | 4.074608 |
|  | Entropy_ph | 1.50682 | 1.96433 | -1.98281 |
|  | Entropy_sir | 1.20014 | -2.43656 | -0.53744 |
|  | Gabor_ph | 0.07984 | -0.06236 | -0.1117 |
|  | Gabor_sir | -0.085 | 0.111228 | 0.10126 |
|  | Info Meas1_ph | -0.1352 | 0.675782 | -0.24977 |
|  | Info Meas1_sir | 0.12777 | 0.235886 | 0.471895 |
|  | Info Meas2_ph | -0.8357 | 0.557415 | 0.293982 |
|  | Info Meas2_sir | 0.36221 | 0.981438 | -0.11777 |
|  | Inverse Difference Moment_ph | 2.51235 | 1.488451 | -0.44691 |
|  | Inverse Difference Moment_sir | 0.94972 | -0.39215 | -1.72053 |
|  | Sum Average_ph | -0.2489 | 0.442019 | 1.445709 |
|  | Sum Average_sir | 0.63978 | 0.760079 | -0.07606 |
|  | Sum Entropy_ph | 0.84754 | -2.19042 | -2.93634 |
|  | Sum Entropy_sir | -3.4428 | 1.624043 | 6.033163 |
|  | Sum Variance_ph | 0.03547 | -0.25562 | -0.03139 |
|  | Sum Variance_sir | -0.5988 | -0.97964 | -1.37346 |
|  | Variance_ph | -0.0363 | -0.18666 | 0.96231 |
|  | Variance_sir | -0.078 | 0.091542 | -0.39858 |

**Supplementary Table 1: A list of 41 features along with a set of example canonical covariates used for LDA based classification of s-p dual stained MSCs after 24 hours of BA, AD, OS and CH (Fig 4b).**

| Predictor | Portion | Rank |
| --- | --- | --- |
| Mean Intensity_sir | 0.3195 | 1 |
| Integrated Intensity_sir | 0.1389 | 2 |
| Difference Entropy_sir | 0.0379 | 3 |
| Info Meas2_sir | 0.0331 | 4 |
| Info Meas1_sir | 0.0286 | 5 |
| Entropy_sir | 0.0286 | 6 |
| Integrated Intensity_ph | 0.0254 | 7 |
| Area | 0.0226 | 8 |
| Sum Entropy_sir | 0.0214 | 9 |
| Max Feret Diameter | 0.0192 | 10 |
| Manders_Correlation | 0.0179 | 11 |
| Info Meas1_ph | 0.0174 | 12 |
| Correlation_sir | 0.0173 | 13 |
| Angular Second Moment_sir | 0.0167 | 14 |
| Solidity | 0.016 | 15 |
| Mean Intensity_ph | 0.0154 | 16 |
| Compactness | 0.0154 | 17 |
| Info Meas2_ph | 0.0147 | 18 |
| Correlation_ph | 0.0145 | 19 |
| Difference Variance_sir | 0.0141 | 20 |
| Major Axis Length | 0.0139 | 21 |
| Min Feret Diameter | 0.012 | 22 |
| Minor Axis Length | 0.0118 | 23 |
| Contrast_sir | 0.011 | 24 |
| Sum Average_sir | 0.0102 | 25 |
| Difference Variance_ph | 0.0099 | 26 |
| Variance_sir | 0.0098 | 27 |
| Inverse Difference Moment_sir | 0.0093 | 28 |
| Gabor_sir | 0.0086 | 29 |
| Sum Variance_sir | 0.0075 | 30 |
| Variance_ph | 0.0075 | 31 |
| Perimeter | 0.0072 | 32 |
| Sum Variance_ph | 0.0063 | 33 |
| Sum Average_ph | 0.0062 | 34 |
| Gabor_ph | 0.0059 | 35 |
| Difference Entropy_ph | 0.0059 | 36 |
| Inverse Difference Moment_ph | 0.0056 | 37 |
| Contrast_ph | 0.005 | 38 |
| Sum Entropy_ph | 0.0049 | 39 |
| Angular Second Moment_ph | 0.0036 | 40 |
| Entropy_ph | 0.0033 | 41 |

**Supplementary Table 2: Predictor screening analysis to show the contribution of individual features for lineage classification. Data shown only for 24 hr timepoint (Fig 4b). SA intensity and texture features were in the top 5, but not the shape descriptors for parsing the cells**

**Supplementary Videos**

**Supp video 1: Time-lapse video showing a reduction in actin turnover during adipogenic (AD) induction as seen by slower decay of SA intensity in the pre-stained MSCs compared to the cells grown in basal media (BA) [ data shown in Fig 3a]**

**Supp video 2:  Time-lapse video showing a faster increase in SA intensity in the unstained MSCs in adipogenic media due to slow down in actin turnover compared to the cells grown in basal media (BA)   [data shown in Fig 3b].**
